# Fractional non ablative 675 nm diode laser versus low fluence high frequency Q- switched 1064 nm nd: YAG laser in the treatment of melasma: a comparative split face trial

**DOI:** 10.1007/s10103-025-04679-2

**Published:** 2025-10-15

**Authors:** Hadeer M. Bassiony, Zakaria M. Obaid, Shady M. Ibrahim, Hany O. Aboelwafa, Mohamed L. Elsaie

**Affiliations:** 1 Department of Dermatology and Venereology, Kafr Elsheikh general hospital, Kafr Elsheikh, Egypt; 2 Department of Dermatology, Venereology and Andrology, Al Azhar University, Damietta, Egypt; 3 Al Azhar University, Cairo, Egypt; 4 Department of Dermatology, National Research Centre, Giza, Egypt

**Keywords:** Melasma, Diode laser, MASI, Fractional laser

## Abstract

Melasma is a chronic, relapsing pigmentary disorder that poses a significant therapeutic challenge, particularly in individuals with darker skin types. Laser therapies have shown promise in targeting both epidermal and dermal melanin deposits. The study aimed to compare the efficacy and safety of fractional non-ablative 675 nm diode laser and Q-switched 1064 nm Nd:YAG laser in the treatment of facial melasma using a split-face design. Thirty female patients with bilateral facial melasma were enrolled in a prospective, split-face trial. Each received five treatment sessions at two-week intervals: the right side with 675 nm diode laser and the left with 1064 nm Q-switched Nd:YAG laser. Clinical efficacy was assessed using the Melasma Area and Severity Index (MASI), dermoscopic scoring, and post-therapy lightening grade at baseline and 12 weeks post-treatment. Both modalities resulted in significant reductions in MASI and dermoscopic scores (p < 0.05). The 675 nm laser showed superior improvement, with a greater proportion of patients achieving excellent lightening. Adverse events were mild and transient. Fractional non-ablative 675 nm diode laser is more effective and equally safe compared to Q-switched 1064 nm Nd:YAG laser in treating facial melasma, offering a promising modality for patients with recalcitrant hyperpigmentation.

**Graphical Abstract:**

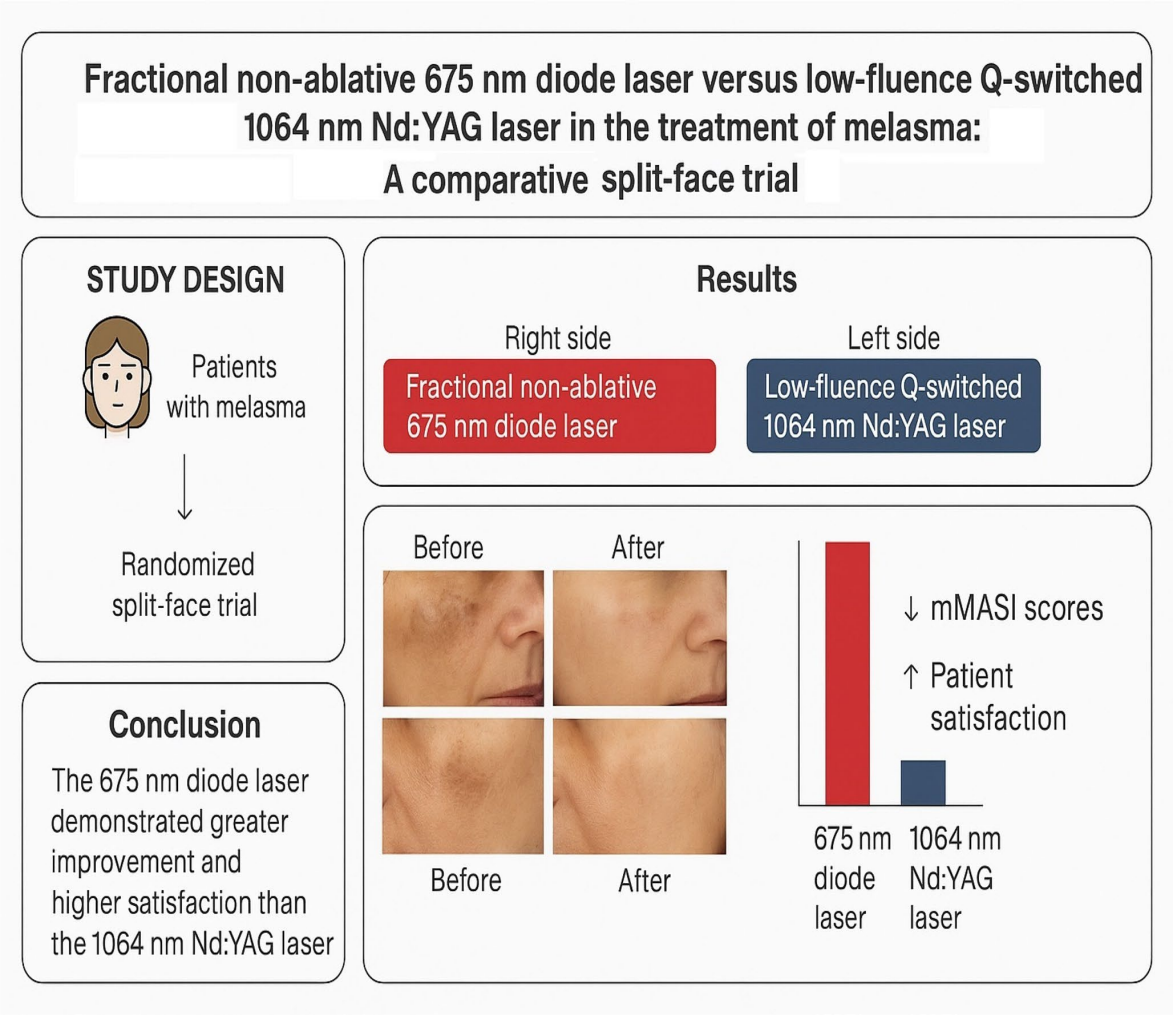

## Introduction

Melasma is a chronic, relapsing hyperpigmentation disorder that primarily affects sun-exposed areas of the face and is especially prevalent among women with darker skin phototypes (Fitzpatrick III–V). Characterized by symmetrical brown macules and patches, melasma significantly impairs quality of life due to its cosmetic impact and tendency to recur despite treatment. Although its pathogenesis is multifactorial, key contributing factors include ultraviolet radiation, hormonal fluctuations, genetic predisposition, inflammation, and vascular changes [1]. 

Traditional treatment approaches—such as topical depigmenting agents, chemical peels, and oral tranexamic acid—are often limited by delayed onset of action, incomplete response, and recurrence [2]. In recent years, laser- and light-based therapies have gained attention for their ability to target deeper dermal pigment while minimizing surface damage. Among these, the Q-switched 1064 nm Nd: YAG laser has been widely utilized due to its deeper penetration and selective photothermolysis of melanin. However, treatment outcomes are inconsistent and often associated with adverse effects, particularly in patients with darker skin tones [3]. 

The fractional non-ablative 675 nm diode laser represents a newer modality that selectively targets melanin and collagen with minimal thermal injury. Its intermediate wavelength offers favorable absorption by melanin with reduced risk of post-inflammatory hyperpigmentation [4]. 

This study aimed to compare the clinical efficacy and safety of the fractional non-ablative 675 nm diode laser and low-fluence, high-frequency Q-switched 1064 nm Nd: YAG laser in the treatment of facial melasma, using a split-face design to control for patient-specific variables.

## Patients and methods

### Study design

This prospective, comparative split-face clinical trial was conducted at Al-Azhar University Hospitals to evaluate the efficacy and safety of fractional non-ablative 675 nm diode laser versus low-fluence, high-frequency Q-switched 1064 nm Nd: YAG laser in the treatment of facial melasma. The study included thirty female patients aged between 20 and 50 years with Fitzpatrick skin types II to V and moderate to severe bilateral facial melasma.

###  Ethical Considerations

The study was approved by an ethics committee of Faculty of Medicine (DFM-IRB 00012367-24-02-001), AL-Azhar University, Damietta; Egypt in accordance with the principles of the Declaration of Helsinki. Confidentiality of patient data was maintained throughout the study. All patients signed both Informed Consent Form and Consent for Image Use.

####  Eligibility Criteria

Patients were enrolled based on specific inclusion and exclusion criteria. Inclusion criteria comprised adult female patients aged 20 to 50 years with Fitzpatrick skin types II–V and bilateral moderate to severe facial melasma. Exclusion criteria included age below 20 or above 50, pregnancy or lactation, systemic conditions associated with hyperpigmentation such as Addison’s disease, use of photosensitizing drugs or hormonal therapies, and prior melasma treatment within three months preceding the study. Diagnosis was established through clinical assessment and confirmed using Wood’s lamp examination, which helped classify melasma as epidermal, dermal, or mixed based on fluorescence enhancement. Only patients with symmetric facial involvement and clear classification were enrolled. All participants agreed to abstain from other topical, procedural, or systemic treatments for melasma throughout the study duration and to adhere to strict photoprotection using sunscreen and protective measures.

### Intervention protocol

Each patient received five laser treatment sessions at two-week intervals. The right side of the face was treated with the fractional non-ablative 675 nm diode laser (Red touch, Deka M.E.L.A., Calenzano, Italy) using settings of 5–7 W power, 100–150 ms dwell time, 1500–2000 μm spacing, and 1–4 passes per session. The left side was treated with a nanosecond Q-switched 1064 nm Nd: YAG laser (Curas; Iloda; South Korea) with a pulse duration of approximately 10 nanoseconds, a 7-mm spot size, 1.0–1.5 J/cm² fluence, and 10 Hz frequency, with up to 4 passes per session. An integrated air-cooling system was employed throughout the procedure to enhance patient comfort and minimize epidermal heating. Laser parameters were adjusted according to individual skin type. Patients were advised to avoid all depigmenting agents during the study and to use a broad-spectrum sunscreen (SPF 50+) and a soothing agent post-treatment.

### Outcome measures

Clinical evaluations were performed at baseline and at 12 weeks after the final treatment session. Clinical evaluation was performed using the hemi-Melasma Area and Severity Index (hemi-MASI) score, which quantifies melasma severity separately for each side of the face. The hemi-MASI score and dermoscopic scoring were performed independently by two blinded dermatologists considering three key domains: area of involvement (A: 0–6), darkness of pigmentation (D: 0–4), and homogeneity of pigmentation (H: 0–4). The hemi-MASI score is calculated using the formula: hemi-MASI = A × (D + H). Each patient’s right and left malar regions were evaluated independently at baseline and 12 weeks post-treatment by two blinded dermatologists. Digital photographs (iPhone 13 Pro Max) were used for objective scoring [5]. Dermoscopic evaluations were carried out using a 3Gen DermLite IV DL4 polarized dermoscope at fixed anatomical landmarks. Pigmentary and vascular features were scored from 0 to 3 (0 = not detected, 3 = obvious). A total dermoscopic score was calculated at baseline and post-treatment. Degree of post-therapy lightening was also assessed based on clinical and dermoscopic examination and graded as none (0%), poor (1–24%), fair (25–49%), good (50–74%), or excellent (75–100%).

### Additional data collection

Additional demographic and clinical data collected included age, occupation, marital status, family history of melasma, sun exposure habits, pregnancy history, and use of hormonal contraceptives.

### Statistical analysis

Statistical analysis was conducted using SPSS version 22. Quantitative data were expressed as mean ± standard deviation and assessed for normality using the Kolmogorov–Smirnov test. Qualitative data were summarized as frequencies and percentages. Paired t-tests or Wilcoxon signed-rank tests were used to compare pre- and post-treatment scores within groups, and chi-square tests were used for categorical comparisons. Spearman correlation was used to explore relationships between continuous variables. A p-value less than 0.05 was considered statistically significant.

## Results

A total of 30 female patients with a mean age of 41.86 ± 5.19 years and mean weight of 80 ± 9.78 kg were enrolled in this study. Half of the participants were employed, 10% reported smoking, and the majority (90%) were married. Fitzpatrick skin types were distributed as follows: type II (6.66%), type III (46.66%), type IV (36.66%), and type V (10%). The predominant melasma type was epidermal (56.66%), with the remainder classified as mixed (43.33%). More than half of the participants (53.33%) had a positive family history of melasma, 83.33% had significant sun exposure, and 80% had a history of pregnancy. Use of hormonal contraceptives was reported in 53.33% of patients. Table 1.

**Table 1:** Demographic and clinical characteristics

Characteristic	Value
Age (years)	**41.86 ± 5.19**
Weight (kg)	**80 ± 9.78**
Occupation (employed)	**15 (50%)**
Smoking	**3 (10%)**
Fitzpatrick Skin Type II	**2 (6.66%)**
III	**14 (46.66%)**
IV	**11 (36.66%)**
V	**3 (10%)**
Melasma Type (Epidermal)	**17 (56.66%)**
Melasma Type (Mixed)	**13 (43.33%)**
Family History	**16 (53.33%)**
Sun Exposure	**25 (83.33%)**
Pregnancy History	**24 (80%)**
Hormonal COC Use	16 (53.33%)

Note: Values expressed as mean ± SD or n (%)

*COC *combined oral contraceptives

**Fig. 1:**
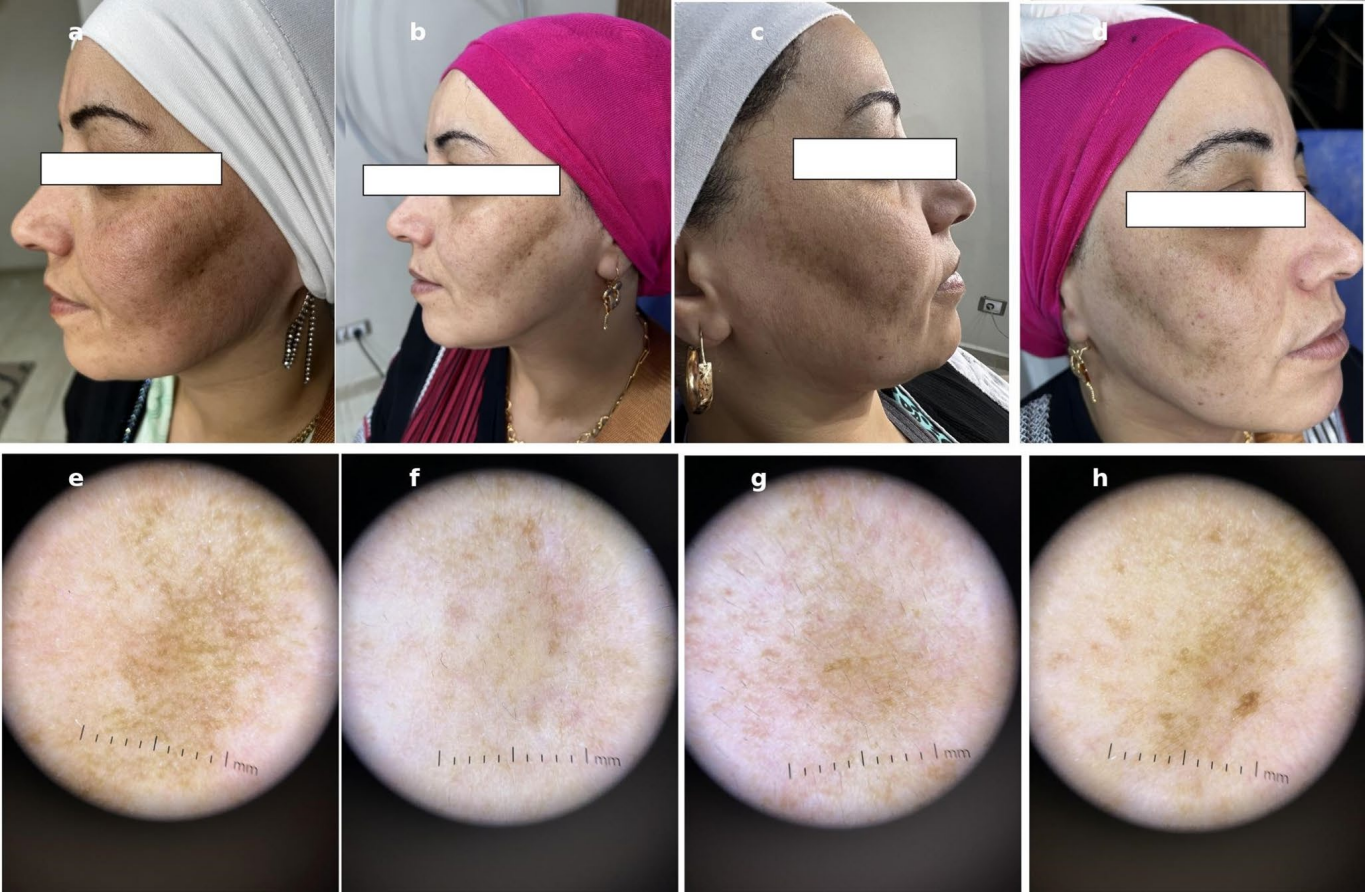
Clinical and dermoscopic images of a female melasma patient before and 12 weeks after split-face laser treatment. The right cheek (a–b), treated with 675 nm diode laser, showed a marked reduction in hemi-mMASI score and improved pigmentation clearance comparFed to the left side. The left cheek (c–d), treated with Q-switched 1064 nm Nd: YAG laser, showed only mild to moderate improvement. Dermoscopy of the right cheek (e–f) revealed initial diffuse light-to-dark brown pseudoreticular pigmentation, multiple brown dots, and prominent telangiectasia, which significantly faded post-treatment. In contrast, the left cheek dermoscopy (g–h) showed persistence of background pigmentation and less pronounced vascular regression

**Fig. 2:**
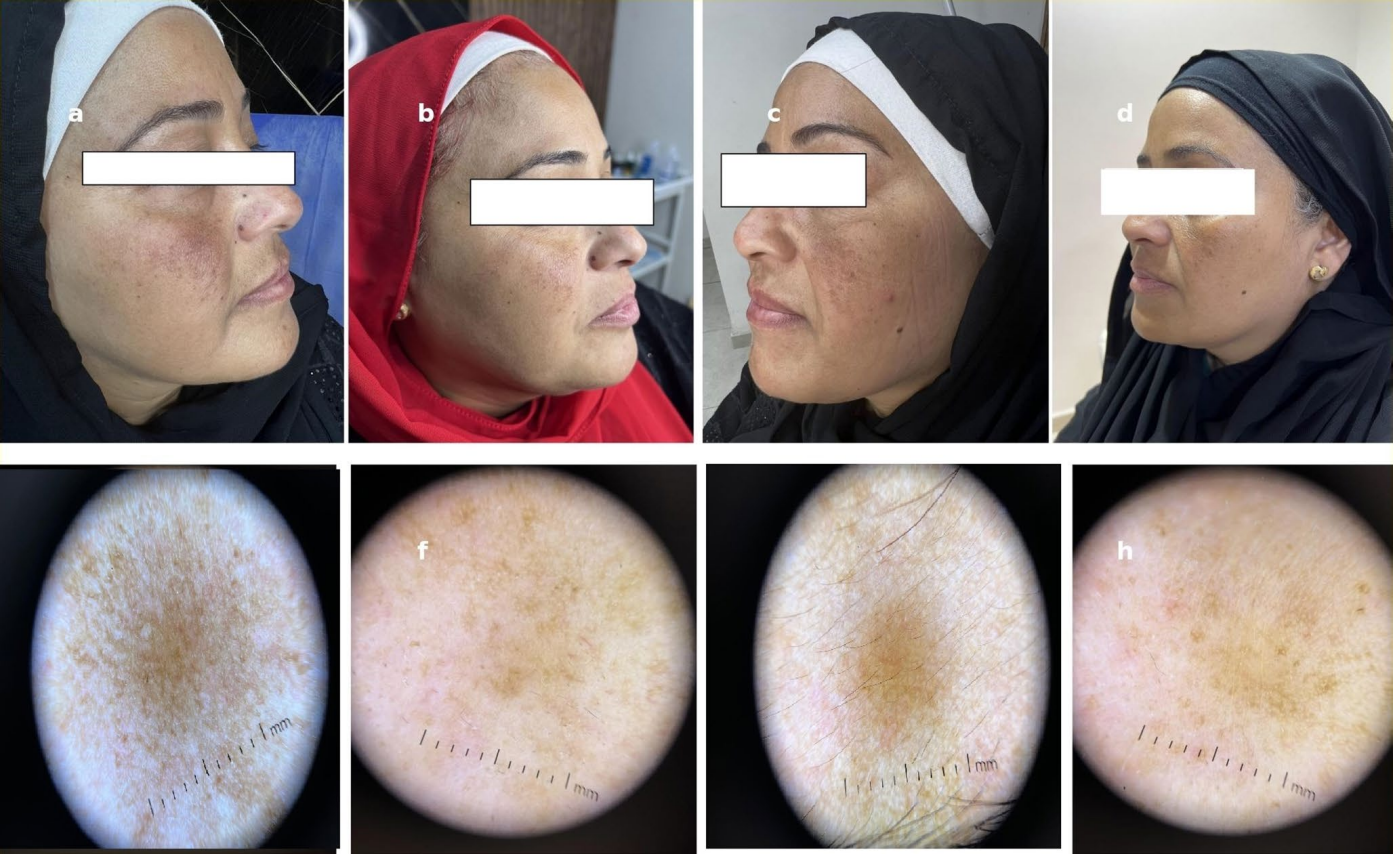
Clinical and dermoscopic images of a female melasma patient before and after 12 weeks of split-face treatment. Right cheek (a–b) was treated with 675 nm diode laser and showed a marked decrease in hemi-mMASI score with improved pigmentation clearance. Left cheek (c–d) was treated with Q-switched 1064 nm Nd: YAG laser, with only partial improvement. Dermoscopy of the right side (e–f) showed pre-treatment brown reticular pigment network and telangiectasia that significantly faded post-treatment. Dermoscopy of the left cheek (g-h) revealed modest pigmentation with vascular regression

There was a statistically significant reduction in the Melasma Area and Severity Index scores on both sides of the face following treatment. The right side, treated with the 675 nm diode laser, showed a greater improvement, with the mean MASI score decreasing from 5.03 ± 1.69 at baseline to 3.33 ± 1.27 post-treatment (*p* = 0.001). The left side, treated with the Q-switched 1064 nm Nd: YAG laser, showed a smaller but still statistically significant reduction from 4.80 ± 1.65 to 3.92 ± 1.65 (*p* = 0.04). Similarly, dermoscopic scores significantly decreased on both sides post-treatment. The right side showed a reduction from 5.06 ± 0.86 to 3.56 ± 0.85 (*p* = 0.001), while the left side decreased from 5.06 ± 0.86 to 3.93 ± 0.86 (*p* = 0.001). Table 2; Figs. 1 and 2.

**Table 2:** MASI and dermoscopic scores before and after treatment

Side	Baseline MASI	Post-treatment MASI	MASI *p*-value	Baseline Dermoscopic Score	Post-treatment Dermoscopic Score	Dermoscopic *p*-value
Right (675 nm)	**5.03 ± 1.69**	**3.33 ± 1.27**	0.001*	**5.06 ± 0.86**	**3.56 ± 0.85**	0.001*
Left (Nd: YAG)	**4.80 ± 1.65**	**3.92 ± 1.65**	0.04*	**5.06 ± 0.86**	**3.93 ± 0.86**	0.001*

Note: MASI: modified Melasma Area and Severity Index

*SD* standard deviation

*p < 0.05 indicates statistical significance

In terms of post-therapy lightening, the right side demonstrated superior outcomes: 36.66% of patients experienced excellent improvement and 53.33% had good improvement. In contrast, the left side showed no excellent outcomes; 50% of patients had fair improvement and 40% had good results. The difference in post-therapy lightening between the two sides was statistically significant (*p* = 0.001). No significant differences were noted between the two sides in baseline MASI and dermoscopic scores. Table 3A positive correlation was found between the MASI score on the right side and being a housewife (*r* = 0.419, *p* = 0.021), while no significant correlations were observed on the left side. Tables 4 and 5;

**Table 3:** Post-Therapy lightening grades

Lightening Grade	Right Side (675 nm)	Left Side (Nd: YAG)	*p*-value
Excellent	**11 (36.66%)**	**0 (0%)**	
Good	**16 (53.33%)**	**12 (40%)**	
Fair	**1 (3.33%)**	**15 (50%)**	
Poor	**2 (6.66%)**	**3 (10%)**	0.001*

Note: Lightening classified by percentage improvement: Excellent (75–100%), Good (50–74%), Fair (25–49%), Poor (1–24%)

*p < 0.05

**Table 4:** Comparison between right and left sides Post-Treatment

Parameter	Right Side	Left Side	*p*-value
Post-treatment MASI	**3.33 ± 1.63**	**3.92 ± 1.65**	**0.16**
Post-treatment Dermoscopic Score	**3.56 ± 0.85**	**3.93 ± 0.86**	**0.09**
Post-Therapy Lightening	**Excellent (36.66%)**	**Fair (50%)**	0.001*

Note: Post-treatment outcomes by side

*MASI* modified Melasma Area and Severity Index

**Table 5:** Correlation between clinical factors and MASI scores (Right vs. Left)

Variable	Right Side *r*	Right Side *p*-value	Left Side *r*	Left Side *p*-value
Age	**0.017**	**0.929**	**0.000**	**1.000**
Sun Exposure	**−0.083**	**0.664**	**−0.012**	**0.949**
Occupation (housewife)	0.419*	**0.021**	**0.304**	**0.102**
Hormonal COC Use	**0.332**	**0.073**	**0.359**	**0.051**

Note: Correlation based on Spearman’s rho. MASI: Melasma Area and Severity Index. *p < 0.05 considered statistically significant; COC: combined oral contraceptives

Both treatment modalities were generally well tolerated, with reported side effects being mild and transient in nature. Erythema was the most commonly observed reaction, occurring in 13.3% of patients on the 675 nm diode-treated side and 20% on the Nd: YAG-treated side. Burning sensation, dryness, and itching were slightly more frequent with the Nd: YAG laser. Notably, one case (3.3%) of post-inflammatory hyperpigmentation (PIH) was reported on the Nd: YAG-treated side, whereas no cases were observed on the 675 nm diode-treated side. Table 6 These findings suggest that while both lasers have acceptable safety profiles, the 675 nm diode laser may offer a more favorable adverse effect profile, particularly in patients with darker skin types prone to pigmentary complications.

**Table 6:** Reported side effects by treatment side

Side Effect	Right Side (675 nm)	Left Side (Nd: YAG)
Erythema	**4 (13.3%)**	**6 (20%)**
Burning Sensation	**2 (6.6%)**	**4 (13.3%)**
Dryness	**1 (3.3%)**	**3 (10%)**
Itching	**1 (3.3%)**	**2 (6.6%)**
Post-inflammatory Hyperpigmentation (PIH)	**0 (0%)**	**1 (3.3%)**

Note: Side effects reported were mild and transient. PIH: post-inflammatory hyperpigmentation. Values represent n (%)

## Discussion

Melasma is a chronic and often recurrent pigmentary disorder predominantly affecting women, particularly those with darker skin phototypes (III–V), and poses a significant therapeutic challenge due to its complex pathogenesis and frequent relapses. Although various treatment modalities have been investigated, laser therapies have gained substantial attention in recent years for their potential to target dermal and epidermal pigmentation components [6]. In this split-face clinical trial, we compared the efficacy and safety of fractional non-ablative 675 nm diode laser with Q-switched 1064 nm Nd: YAG laser in the treatment of facial melasma. Our findings demonstrated a statistically significant greater reduction in MASI scores and higher patient satisfaction with the 675 nm diode laser compared to the Q-switched 1064 nm Nd: YAG laser. These results underscore the promising role of the 675 nm laser as a novel treatment modality for melasma [3]. 

The greater efficacy of the 675 nm diode laser may be attributed to its unique mechanism of action. Unlike traditional lasers used in melasma management, the 675 nm wavelength selectively targets melanin chromophores with a lower risk of collateral damage to surrounding tissues [7]. It achieves this through controlled non-ablative thermal stimulation of the dermis and epidermis, which may enhance dermal remodeling and reduce melanogenesis without disrupting the epidermal barrier. This laser also stimulates fibroblast activity, leading to the production of new collagen and matrix remodeling, which can indirectly modulate the melanogenic environment and mitigate the pigmentary process [8]. 

Additionally, the 675 nm laser exerts its effects through photobiomodulation, which improves skin texture and tone by promoting mitochondrial activity, reducing oxidative stress, and modulating inflammatory pathways [9]. Since inflammation and oxidative stress are known contributors to melasma pathogenesis, the anti-inflammatory and antioxidant effects of the 675 nm laser could explain the observed clinical improvements. [10, 11].

In contrast, the Q-switched 1064 nm Nd: YAG laser has long been employed in melasma treatment due to its ability to penetrate deeply into the dermis and selectively target melanin via a photoacoustic effect [12]. – [13] Despite its efficacy in reducing pigmentation, it carries a risk of post-inflammatory hyperpigmentation (PIH), particularly in individuals with darker skin tones, and often requires multiple treatment sessions for satisfactory results [14, 15] In our study, although the Nd: YAG laser produced a significant improvement in MASI scores from baseline, it was less effective than the 675 nm diode laser, and patient satisfaction scores were comparatively lower. This aligns with previous studies reporting variable outcomes and a high relapse rate following Nd: YAG laser therapy, particularly when used as monotherapy [14, 16]. 

Importantly, both lasers were well tolerated in our study, with minimal side effects reported and no serious adverse events. Erythema and mild burning sensation were the most common transient adverse effects in both groups, resolving within hours to days. Notably, the 675 nm diode laser group reported fewer pigmentary side effects, suggesting a superior safety profile in darker skin types. This is a significant finding given that melasma patients often seek treatment not only for cosmetic reasons but also to improve quality of life, and side effects such as PIH can be psychologically distressing and counterproductive to treatment goals.

Our results are consistent with and build upon the limited existing literature on the use of 675 nm lasers in pigmentation disorders. Campolmi et al. [7] and Cannarozzo et al. [8] reported favorable outcomes in photoaging and lentigines using the 675 nm diode laser, with improvements in pigmentation, skin texture, and dermal collagen content. Although these studies were not conducted specifically in melasma patients, the underlying principles of selective photothermolysis and dermal remodeling are applicable. Furthermore, Cannarozzo et al. [9] emphasized the 675 nm laser’s ability to achieve clinical improvement without causing thermal damage to the epidermis—a particularly beneficial characteristic in melasma therapy where epidermal injury often precipitates pigmentary rebound.

Another critical aspect of melasma management is the chronicity of the condition and the frequent recurrence following cessation of therapy. One hypothesis is that dermal and vascular components contribute to disease persistence and resistance to epidermal-targeted treatments [10]. The 675 nm laser’s non-ablative and dermally-focused approach may help address this limitation by modulating the dermal environment. This could include vascular normalization, fibroblast reprogramming, and reduction of mast cell activity, all of which have been implicated in melasma pathology [17]. This suggests that the 675 nm laser may not only improve pigmentation but also affect the underlying pathophysiology, potentially leading to longer remission periods.

While our study adds meaningful data to the field, it is essential to recognize its limitations. First, the follow-up period was limited to 3 months post-treatment. Longer follow-up is needed to assess the durability of the treatment response and the potential for recurrence. Second, although the split-face design controls for individual variability in melasma severity and skin type, a larger sample size across different Fitzpatrick phototypes would provide more generalizable data. Third, we did not perform histopathological or immunohistochemical analyses to corroborate the clinical findings with biological markers of melanogenesis or dermal remodeling. Minor variations in lighting, background, and patient positioning may have affected the before-and-after photographs. However, objective scoring by blinded dermatologists minimized the impact of these inconsistencies. Future studies incorporating skin biopsies and molecular assays could provide mechanistic insights into the observed clinical outcomes.

Additionally, combining laser therapy with topical agents such as hydroquinone, tretinoin, or tranexamic acid might enhance results and prevent recurrence. Several studies have demonstrated that lasers, when used in combination with topical depigmenting agents or systemic therapies, produce more durable and profound responses [18, 19]. It remains to be determined whether the 675 nm diode laser may serve better as a monotherapy or as part of a multimodal regimen, especially for recalcitrant or mixed-type melasma.

Patient selection also remains a cornerstone of laser-based melasma management. Factors such as disease chronicity, depth of pigmentation, hormonal influences, and lifestyle behaviors (e.g., sun exposure, use of hormonal contraceptives) play important roles in treatment outcomes. While both lasers tested in this study were effective to varying degrees, tailoring therapy based on individual patient characteristics may yield more personalized and successful outcomes. Future studies could focus on developing predictive markers or algorithms to identify which patients are more likely to benefit from specific laser treatments.

Another promising area for research is the potential of fractional and low-fluence lasers to induce subclinical injury that promotes gradual pigment clearance while minimizing inflammation [8]. This concept underpins the rationale behind the use of fractional devices, such as the 675 nm diode laser employed in this study, which create controlled microthermal zones and spare surrounding tissue [3]. By avoiding widespread thermal damage, these devices promote wound healing and melanocyte regulation through regenerative rather than destructive mechanisms [11]. 

Moreover, advancements in laser technology, such as real-time feedback systems, customized pulse durations, and integration with cooling mechanisms, may further improve treatment precision and reduce complications. These technological refinements are particularly important in melasma management, where even minor side effects can lead to significant patient dissatisfaction and relapse [3]. 

From a patient-centered perspective, the higher satisfaction scores with the 675 nm diode laser in our study reflect not only its clinical efficacy but also the comfort and tolerability of the procedure [2]. Patient-reported outcomes, often overlooked in clinical trials, are essential in chronic dermatological conditions like melasma, where quality of life and self-esteem are closely tied to treatment success. The minimal downtime, fewer adverse effects, and visible improvement associated with the 675 nm laser likely contributed to the favorable patient perception [7, 8].

In conclusion, our findings support the use of fractional non-ablative 675 nm diode laser as a safe and effective modality for treating facial melasma, with superior clinical outcomes and patient satisfaction compared to the Q-switched 1064 nm Nd: YAG laser. Its favorable safety profile, particularly in darker skin types, makes it a promising addition to the therapeutic armamentarium for melasma. Future large-scale, long-term studies are warranted to confirm these results and explore its role in combination regimens and maintenance therapy. 

## Data Availability

The data that support the findings of this study are available from the corresponding author upon reasonable request.

## Notes

All authors confirm all data and materials as well as software applications or custom code support their published claims and comply with field material.

## Abbreviations

mMASI: Modified Melasma Area and Severity Index
PIH: Post-Inflammatory Hyperpigmentation
COC: Combined Oral Contraceptive
Nd:YAG: Neodymium-Doped Yttrium Aluminum Garnet
SPF: Sun Protection Factor
SD: Standard Deviation
r: Spearman Correlation Coefficient

## References

[1] AboAlsoud ES, Eldahshan RM, AbouKhodair Mohammed H, Elsaie ML (2022) Safety and efficacy of topical Metformin 30% cream versus triple combination cream (Kligman’s formula) in treating melasma: a randomized controlled study. J Cosmet Dermatol 21(6):2508–2515 35357753 10.1111/jocd.14953

[2] Atwa MA, Ahmed AH, Nada HA, Refaey SM, Jafferany M, Elsaie ML (2022) Combined chemical peels versus trichloroacetic acid (TCA) for treating melasma: a split face study. J Dermatolog Treat 33(2):959–964 32649234 10.1080/09546634.2020.1793888

[3] Zhang B, Xie B, Shen Y, Zhang L, Song X (2022) Single and combined 1064 nm Q-switched nd: YAG laser therapy in melasma: a meta-analysis. J Cosmet Dermatol 21(9):3794–3802 35876484 10.1111/jocd.15270

[4] Ibrahim SMA, Elsaie ML, Fusco I, Zingoni T, Rageh MA (2025) A 675 nm laser in the treatment of facial melasma in dark skin types. Photobiomodul Photomed Laser Surg 43(3):90–95 39895335 10.1089/photob.2024.0102

[5] Pandya AG, Hynan LS, Bhore R, Riley FC, Guevara IL, Grimes P et al (2011) Reliability assessment and validation of the melasma area and severity index (MASI) and a new modified MASI scoring method. J Am Acad Dermatol 64(1):78–83 e1–2 20398960 10.1016/j.jaad.2009.10.051

[6] Zaky MS, Obaid ZM, Khalil EA, Elsaie ML (2021) Microneedling-assisted topical tranexamic acid solution versus 4% hydroquinone for treating melasma: a split-face randomized study. J Cosmet Dermatol 20(12):4011–4016 34525492 10.1111/jocd.14440

[7] Campolmi P, Bonan P, Cannarozzo G, Bruscino N, Rossi R, Lotti T (2020) Non-ablative fractional laser resurfacing in acne scarring. J Clin Aesthet Dermatol 13(7):27–33

[8] Cannarozzo G, Tamburi F, Lombardi R, Bonan P (2020) Use of a 675-nm laser in the treatment of facial photoaging: a clinical study. J Cosmet Laser Ther 22(5):268–272

[9] Cannarozzo G, Negosanti F, Sannino M, Bennardo L, Tamburi F, Bonan P (2021) A novel 675 Nm laser device in the treatment of facial melasma: a pilot study. J Cosmet Dermatol 20(4):1085–1090

[10] Kim M, Jung YJ, Park HJ (2017) Pathophysiology and new treatments for melasma. Int J Mol Sci 18(4):82428406445

[11] Wang JV, Albornoz CR, Au SC, Saedi N (2019) Advances in laser treatments for melasma. J Clin Aesthet Dermatol 12(10):30–3631360286

[12] Cho SB, Lee SJ, Kang JM, Kim YK, Lee JH (2009) The efficacy and safety of low-fluence Q-switched Nd:YAG laser for the treatment of melasma in Asian patients. Lasers Med Sci 24(5):645–650

[13] Elsaie ML, Hussein MS, Tawfik AA, Emam HM, Badawi MA, Fawzy MM, Shokeir HA (2016) Comparison of the effectiveness of two fluences using long-pulsed Nd:YAG laser in the treatment of striae distensae. Histological and morphometric evaluation. Lasers Med Sci 31(9):1845–1853 27595152 10.1007/s10103-016-2060-2

[14] Lee HJ, Lee MH, Lee DY (2010) Efficacy and safety of low-fluence 1064-nm Q-switched nd:yag laser treatment for melasma in Asian skin. J Dermatolog Treat 21(3):210–215

[15] Trelles MA, Allones I, Luna R (2010) Treatment of melasma with pulsed CO2 laser: a pilot study. Lasers Med Sci 25(5):679–684

[16] Wattanakrai P, Mornchan R, Eimpunth S (2014) Low-fluence Q-switched nd:yag laser for melasma: a split-face comparative study. J Am Acad Dermatol 70(1):123–129

[17] Kwon SH, Na JI, Choi JY, Park KC (2018) Melasma: updates and perspectives. Exp Dermatol 27(6):704–708 10.1111/exd.13844 30422338

[18] Kong SH, Suh HS, Lee AY (2018) Phototherapy and lasers for melasma. Clin Dermatol 36(1):59–65

[19] Del Rosario E, Florell SR, Ong L, Grossman M, Burris K, Peterson JD (2018) Melasma therapy with combination topical and laser treatments: a systematic review. Dermatol Surg 44(2):257–270

